# Accidental Children Poisoning With Methadone: An Iranian Pediatric Sectional Study

**Published:** 2013

**Authors:** Sayena JABBEHDARI, Fariba FARNAGHI, Seyed Fakhreddin SHARIATMADARI, Narjes JAFARI, Fatemeh-Fereshteh MEHREGAN, Parvaneh Karimzadeh

**Affiliations:** 1 Pediatric Neurology Research Center, Shahid Beheshti University of Medical Sciences, Tehran, Iran; 2 Department of Pediatric, Loghman Hospital, Shahid Beheshti University of Medical Sciences, Tehran, Iran; 3 Pediatric Neurology Department, Mofid Children’s Hospital, Faculty of Medicine, Shahid Beheshti University of Medical Sciences, Tehran, Iran

**Keywords:** Children, Opium, Methadone, Poisoning

## Abstract

**Objective:**

Toxic poisoning with methadone is common in children in Iran. Our study was carried out due to the changing pattern of methadone poisoning in recent years and increasing methadone toxicity.

**Materials & Methods:**

In this descriptive-sectional study, all of the methadone poisoned children younger than 12 years who were admitted to the Loghman Hakim Hospital in 2012, were assessed. Clinical symptoms and signs, para-clinical findings, and treatment were evaluated.

**Results:**

In this study, 16 boys and 15 girls who had been poisoned by methadone were enrolled. The mean age of patients was 55 months. All patients had been poisoned randomly or due to parent’s mistakes. The mean time of symptoms onset after methadone consumption was 1 hour and 30 Min, indicating a relatively long time after onset of symptoms.

Clinical findings were drowsiness (75%), miotic pupil (68 %), vomiting (61%), rapid shallow breathing (57%) and apnea (40%). In paraclinical tests, respiratory acidosis (69%) and leukocytosis (55.2%) were seen. The most important finding was increase in distance of QT in ECG (23.8%). The mean time of treatment with naloxone infusion was 51 hours. Three percent of patients had a return of symptoms after discontinuation of methadone. In patients with apnea, a longer course of treatment was required, and this difference was significant. Also, 17% of patients with apnea had aspiration pneumonia, which was statistically significant.

**Conclusion:**

We suggest long time treatment with naloxone and considering the probability of return of symptoms after discontinuation of methadone.

## Introduction

Poisoning with methadone and its derivatives are very harmful and deadly, and can be the cause of decreased level of consciousness, coma, apnea, respiratory suppression, and death. This poisoning is a very common poisoning in our country, so that approximately 10% of hospitalized patients who were referred to Loghman- Hakim hospital had the complaint of methadone poisoning (1,2). Twenty-five cases of methadone poisoning were diagnosed at Loghman-Hakim hospital from 1991 to 1994, and in 1998 and 2008, respectively 49 and 60 cases of this poisoning were diagnosed. This statistics showed the increasing trend of this harmful poisoning. There are many ways to use methadone and other drugs; consequently, there may be intoxication due to abuse of drugs. Methadone (Dolophine) is a synthetic opioid drug with, long half life and the very good analgesic effect. The half life of the drug is between 25 to 52 hours (2). 

In recent years, consumption of methadone as syrup or tablet for relief of severe pain and addiction to it has been common in Iran. Methadone syrup is an oral solution of 5 mg/mL methadone hydrochloride. Dose of 1 mg/kg can lead to serious apnea and death. Unsafe keeping of this drug in water bottle can be wrong (1). The adverse effects of methadone consumption in children may be nausea, vomiting, malaise, and drowsiness, and without treatment they go into apnea or coma. 

The other side effects of methadone are heart complications and ECG change as increase in QT interval. The available information on symptoms and clinical findings are about adult poisoning with methadone.

## Materials & Methods

This descriptive-sectional study was done on children with methadone poisoning at the Loghman-Hakim hospital in second half of 2012. Other suspected cases of poisoning were excluded. The assessment information of the patients was gathered as age, gender, past medical history, development status, general appearance, and clinical and neuroimaging findings. Quantitative data were shown as mean and qualitative data as percent. The data were analyzed by SPSS version 17. T-test was used for analyzing the quantitative variables, and chi-square and Fisher’s exact tests for qualitative variables. Statistical significance level in this study was considered to be p<0.05.

## Results

Thirty-one patients with methadone poisoning were assessed. The mean age of patients at assessment time was 55 months. Only 19% of patients were under one year of age, and the youngest patients was 4 months. Fifteen patients were girls and 16 patients were boys. The reason of poisoning in all of the patients was accidental oral poisoning with methadone. In 20 patients, the poisoning was assessed and it was found that 12 patients had taken methadone syrup that the minimum dosage of methadone was 2 mg and the maximum dosage was 125 mg (mean dose, 33 mg). Eight patients had used methadone as tablet form, that the minimum dose was 2.5 mg and maximum dose was 25 mg (mean dose, 21 mg).

The mean time of methadone consumption until symptom onset was 1.53 hours and the mean time of symptom onset until admission was 2.4 hours. Thus, there was a relatively long time from consumption to symptom onset. In most cases, the first symptom was drowsiness and then, respiratory depression was detected. These symptoms are very important and life-threatening. The classic triad of opium poisoning includes loss of consciousness, respiratory depression, and miotic pupil that was observed in 62 % of patients. Vomiting was very common in this study. 

There was a significant relationship between apnea (as a very important and critical side effect) and the dosage of methadone. In patients with apnea, the percentages of miotic pupil, seizure, and respiratory infections were very high. All of the pneumonia aspiration and respiratory infections occurred in patients with apnea. In 17% of patients with apnea, pneumonia aspiration was seen, that this result was statistically significant (p<0.022). 

The most common findings in the lab data were respiratory acidosis (69%), leukocytosis (55.2%), and hyponatremia (17%). Six percent of patients had decreased level of glucose and 7% had increased level of glucose. The important finding in ECG was increase in QT interval (23.8%).

Treatment of the patients was done by stat dosage: 0.76 mg (1.9 ampoules) and infusion Dosage: 23 mg (58 ampoules) of naloxone. The mean time of naloxone infusion was 51 hours, and in 3% of patients, the symptoms of poisoning returned after treatment and discontinuation of naloxone. 

In patients with apnea the duration of treatment was longer than patients without apnea (58.5 hours vs 42 hours, respectively), and this difference was statistically significant (p<0.007).

## Discussion

Methadone is synthetic opium with long-acting effect. Unfortunately, the consumption of this drug has increased recently. After popularity of alternative treatment with methadone which began in 2001 and has gradually increased, today more than 1500 MMT centers are active in the country. The increased consumption of methadone and failure to adopt effective measures to prevent poisoning are the causes of toxicity that has become a serious threat to children. 

For example, in Canada, label was made on the container of methadone, but methadone poisoning and death have still been reported (3). In our study, the mean age of the patients was 55 months, which is more than that of by Binchy et al’s study that was done on opium poisoning (4). In our study, the dominant symptoms were decreased level of consciousness, respiratory depression, apnea, miosis, and seizure. This result was in accordance with those of other studies (1,2). Classic triad of methadone poisoning were seen in 62% of cases, and in Zamani’s study which was done on opium poisoning, the triad were seen in 25% of patient (5). In our study, the incidence of methadone poisoning is the same in girls and boys, but in other studies, there are not any reports of this matter (5). In our assessment, duration of the first symptom was 1.53 hours, and the mean time was 51 hours. LoVecchio et al. in their study done on 44 cases of methadone poisoning in patients over 18 years, reported that the duration of the first symptom was 3.2 hours and maximum 9 hours, and the time needed for treatment was 24 hours (6). In another studies, this time was from 6 hours to 48 hours. Changing in the pattern of ECG as bradycardia was noted in various studies, but decrease in QT interval was seen in adult patients (7). In our patients, uncommon symptoms such as delirium and itching occurred, that these symptoms were not reported in other studies.

In recent articles, some of the symptoms, such as ataxia, cerebellitis, hearing loss and chest wall rigidity were reported (8,9). In the present study, the mean time from symptom onset until admission was 2.4 hours, and we had no reports of death among our patients, but in other papers, there are some reports of death among their patients (3,6,10).

**In conclusion, **Methadone is a synthetic opium and poisoning with this drug could be harmful and deadly, and can cause decreased level of consciousness, apnea and coma. Proper maintenance of the drug and keeping out of reach of children is recommended to prevent the risk of toxicity.

## Declaration of conflicting interests

None declared.

## Funding

The authors received no financial support for the research and publication of this article.
